# Primary mucinous cystadenoma of the spermatic cord within the inguinal canal

**DOI:** 10.1186/1746-1596-7-139

**Published:** 2012-10-08

**Authors:** Jee-Yeon Kim, Young-Taek Lee, Hyun-Jeong Kang, Chang-Hun Lee

**Affiliations:** 1 Department of Pathology and Medical Research Institute, Pusan National University Hospital, 1-10 Ami-dong, Seo-gu, Busan, 602-739, Republic of Korea; 2 Department of Surgery, Kwang Hye Hospital, Busan, Republic of Korea; 3 Department of Pathology, Kwang Hye Hospital, Busan, Republic of Korea

**Keywords:** Mucinous cystadenoma, Vas deferens, Spermatic cord

## Abstract

We report a hitherto not documented case of primary mucinous cystadenoma arising in the spermatic cord within the right inguinal canal of a78-year-old man. The tumor was painless, hard and mobile. A computed tomography scan on the pelvis revealed an oval shaped, low attenuation mass, measuring 5.0x2.5x2.1 cm, that was present adjacent to the vas deferens. Grossly, the excised mass was multicystic mucinous tumor, filled with thick mucoid materials. Microscopically, the cystic wall was irregularly thickened. The cystic epithelium commonly showed short papillae lined by a single layer of columnar to cuboidal mucinous epithelial cells without significant stratification or cytologic atypia. Goblet cells were also frequently present. Immunohistochemically, the neoplastic cells showed positive reaction to carcinoembryonic antigen, cytokeratin 20, CDX2, epithelial membrane antigen, and CD15. However, they were negative for PAX8 and Wilms’ tumor 1 protein. Pathological diagnosis was a papillary mucinous cystadenoma of the spermatic cord. Although mucinous cystadenoma in this area is extremely rare, it is important that these lesions be recognized clinically and pathologically in order to avoid unnecessary radical surgery.

**Virtual slides:**

The virtual slide(s) for this article can be found here: http://www.diagnosticpathology.diagnomx.eu/vs/1720965948762004

## Background

Primary tumors of the spermatic cord can be of many types, but cystadenoma is especially exceptional. To the best of our knowledge, this is the third report of primary cystadenoma in the spermatic cord since the original report by McCluggage et al. in 1996 [1]. Interestingly, the neoplastic cells of the present case show mucinous character with intestinal differentiation, which is a unique finding distinct from previous two reports [1,2]. The clinicopathological features of this tumor are presented herein with a brief review of the differential diagnosis, pathology and possible histogenesis.

## Case presentation

### Clinical summary

**Figure 1:**
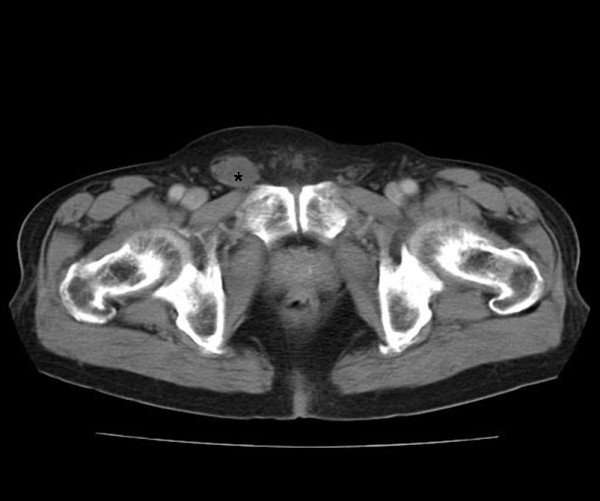
**Contrast-enhanced CT scan on the pelvis.** It reveals an oval shaped, low attenuation mass (asterisk), measuring 5.0x2.5x2.1 cm, and showing a well demarcated smooth margin in the right inguinal area.

A 78-year-old man presented with a painless right inguinal swelling that was firstly noticed 3 months ago. The right inguinal mass was hard mobile. Otherwise, there were no other specific symptoms. During physical examination, spermatic cords could be palpated on both sides. The testis and epididymis appeared normal. Routine blood analysis was all within normal limits. Ultrasound imaging on the right inguinal area showed a lobulated hypoechoic mass, measuring 5.0 cm, without internal vascularity. A computed tomography (CT) scan on the pelvis revealed an oval shaped, low attenuation mass, measuring 5.0x2.5x2.1 cm and showing a well demarcated smooth margin in the right inguinal area (Figure 1). At operation, a white cystic mass was found within the right inguinal canal and adhered to the vas deferens. The tumor was completely excised with an adjacent part of the vas deferens.

### Pathological findings

**Figure 2:**
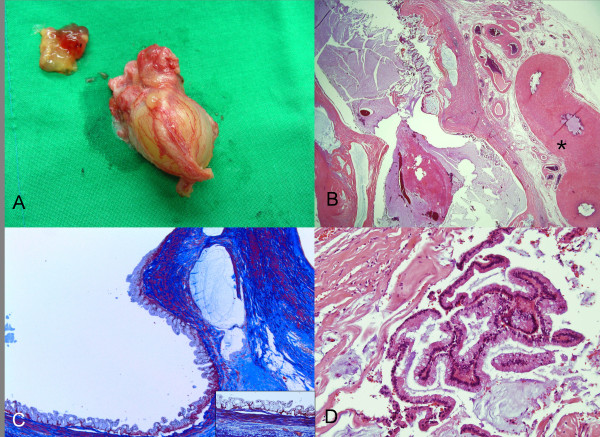
**Gross and microscopic findings of the cystic inguinal tumor.** The tumor is completely excised with an adjacent part of vas deferens. A thick mucin content, taken out of the cystic tumor, is also noted (**A**). A multicystic mucinous tumor, filled with gelatinous mucoid materials, is microscopically present. The cystic wall, neighboring to vas deferens (asterisk), is irregularly thickened and fibrotic (**B**, x20). Masson trichrome stain clearly displays degenerated but still encircling muscular layers in the cystic wall of the tumor (**C**, x40; inlet showing high power view of x200). The cystic epithelial lining shows short papillary formation, and consists of a single layer of bland-looking columnar mucinous epithelial cells with frequent goblet cells (**D**, x 200).

Gross pathological examination revealed a multicystic mucinous tumor, measuring 4.5 cm in greatest diameter, filled with gelatinous mucoid materials (Figure 2A). Microscopically, the cystic wall was irregularly thickened and fibrotic (Figure 2B). The cystic epithelial lining was frequently detached from the wall, and focally showed short simple papillae supported on delicate fibrovascular stalks. The epithelium itself consisted of simple mucinous, nonciliated columnar cells with basally located small nuclei. In the most part of the epithelium, the nuclei are arranged in one or two layers. On Masson trichrome stain, the mucinous cystic tumor clearly displayed degenerated but encircling muscular layers which were reminiscent of muscular coat of the vas deferens (Figure 2C). Intraepithelial goblet cells were frequently present. However, neither remarkable papillary tufting nor nuclear atypia was present. Mitoses were rare or absent (Figure 2D). Areas of mucin extravasation into stroma were present, but there was no stromal invasion by tumor cells. These histologic findings were qualifying the tumor as benign mucinous neoplasm. The vas deferens around the tumor was histologically unremarkable. Its lumen was empty. The tumor was close to the vas deferens, but the direct contiguity between both structures was not definitely noted. On the contrary, adense collagenous tissue separated the tumor from adjacent vas deferens tubules. Spermatozoa were present in neither the tumor nor the vas deferens, possibly reflecting senile atrophy of the testicles.

### Immunohistochemistry

**Figure 3:**
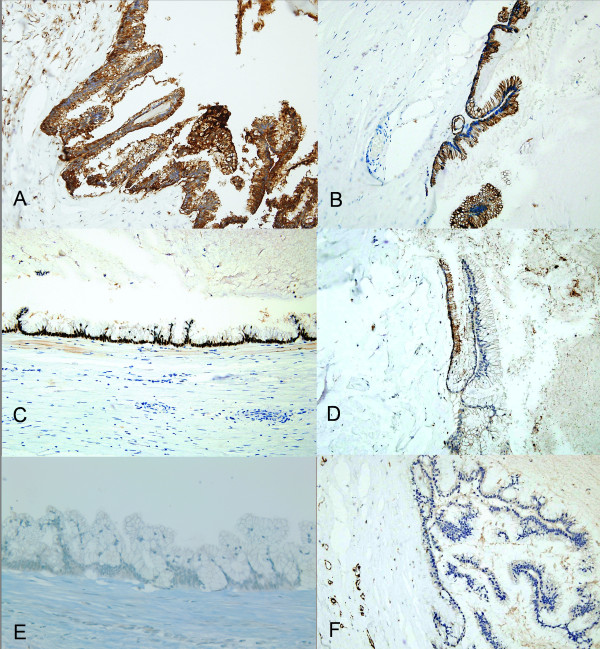
**Immunohistochemical findings of the tumor.** Immunostaining for CEA. The cytoplasm of neoplastic cells shows diffuse strong positive reaction to CEA (**A**, x 200). Immunostaining for CK20. The tumor cells also exhibit diffuse positive reaction to CK20 (**B**, x 200). Immunostaining for CDX2. The nuclei of neoplastic epithelial cells display diffuse strong positive reaction to CDX2 (**C**, x 200). Immunostaining for CD15. The cells are focally positive for CD15 (**D**, x 200). Immunostaining for PAX8. The cells are negative for PAX8 (**E**, x 200). Immunostaining for WT-1. The cells are negative for WT-1 (**F**, x 200).

Immunohistochemically, the neoplastic cells showed diffuse positive staining to carcinoembryonic antigen (CEA; clone II-7; Dako, Glostrup, Denmark), cytokeratin 20 (CK20; clone Ks20.8; Lab Vision Corp., Fremont, CA, USA), CDX2 (clone DAK-CDX2; 1:25, DAKO) and epithelial membrane antigen (EMA; clone E29; Dako). They were also focally positive for CD15 (clone Carb-3; Dako), but negative for PAX8 (clone PAX8R1; Abcam Inc., Cambridge, MA, USA) and Wilms’ tumor 1 protein (WT-1; clone 6 F-H2; Dako) (Figure 3). Adjacent ductus deferens tubule showed diffuse positive staining to EMA, CD15 and PAX8, but negative reaction to CEA, CK20, CDX2 and WT-1. Pathological diagnosis was a papillary mucinous cystadenoma of the spermatic cord.

### Follow-up

After surgery, the patient has recovered well, and shows no recurrence at 8 months’ follow-up after local excision of the tumor.

## Discussion

Primary tumors of the spermatic cord are rare. Furthermore, because of the intimate anatomic connection of the spermatic cord with the scrotum and the tunica vaginalis testis, it is often impossible to decide from which of these anatomic compartments these tumors have arisen. From a topographic and surgical standpoint, it would perhaps be more appropriate to simply divide them into those of the scrotum and those of the inguinal canal, without attributing them to a particular anatomic structure [3]. Topographically, the present case was arisen from the spermatic cord within the inguinal canal outside the epididymis.

Grossly, the tumor was entirely cystic. Microscopically, the epithelium of the cystic wall consisted of simple mucinous, nonciliated columnar cells with basally located small nuclei. The uniform round to oval nuclei lacked atypia and arranged in one or two layers. Focally, the epithelium showed simple short papillae. Characteristically, there was noted the presence of abundant luminal mucin and intraepithelial goblet cells. The findings were indicative of mucinous cystadenoma with intestinal differentiation. The intestinal differentiation was confirmed from positive reaction of the epithelial cells to CEA, CK20 and CDX2. Only 2 previously reported cases of cystadenoma developed in the spermatic cord of the inguinal region have been so far found in the English literature [1,2]. However, the tumors had serous epithelial lining (of benign or borderline character) which was histologically distinguished from the present case. In addition, serous cystic tumors, two-thirds of which seemed to be a partial manifestation of von Hippel-Lindau’s syndrome, have been also described in the head of the epididymis. Approximately, 40% of these epididymic tumors are known to follow bilateral occurrence [4]. And in those cases, the efferent ducts of the epididymis are proposed as the origin of a papillary cystadenoma in the epididymis [5]. Regarding clinical manifestation, there was no evidence of syndromatic pattern and bilaterality in the present case, including his relatives.

As a matter of fact, we were not able to establish the tissue of origin of this papillary mucinous cystadenoma with certainty. However, we could make two speculations. Firstly, because the tumor did not show any direct connection with surrounding vas deferens tubules, we consider its origin from mesonephric (or Wolffian) duct anlages on the basis of its location and microscopic appearance. In the present case, neoplastic mucinous cells revealed cytoplasmic positivity to CD15 and EMA which was similar to immunohistochemical pattern of epithelial cells of normal vas deferens. Furthermore, the tumor was encircled by a rather degenerated, thin smooth muscle coat that was identified from Masson trichrome stain. Taken altogether, the features would be reminiscent of mesonephric duct anlage structure. Additionally, the tumor cells showed diffuse positive reaction to CK20, CDX2 and CEA which is characteristic of intestinal epithelial cells, and frequent occurrence of goblet cells in the epithelium. These findings of intestinal differentiation are those not expected in normal vas deferens, but may be related to versatile phenotypic expression frequently seen in mucinous neoplasms elsewhere. We also performed PAX8 immunohistochemical stain to identify if there is a possible evidence of Müllerian differentiation in the tumor. PAX8 is a member of the paired box (PAX) family of nuclear transcription factors and is important in organogenesis of the thyroid, kidney, and Müllerian system. PAX-8 is known to be expressed in a variety of ovarian epithelial tumors [6]. In the present case, however, the tumor cells were negative for PAX8. Thus, the evidence of Müllerian differentiation was not proven in this case. Secondly, the lesion may be resulted from a post-inflammatory cystic dilatation of a segment of the vas deferens, and followed by intestinal metaplasia and mucin production. In this speculation, a peritumoral fibromuscular wall that was histologically seen may be resulted from any sequelae of chronic inflammation.

It is well known that the histology of mucinous cystadenoma is commonly found in female ovary. Generally, ovarian mucinous cystadenomas are large, unilateral, multilocular cystic tumors containing mucoid material. Microscopically, they are composed of glands and cysts lined by a single layer of columnar cells with abundant intracellular mucin. Cellular stratification is minimal, and nuclei are basally located with only mild atypia. Papillary formation in ovarian mucinous cystadenoma is unusual, but when it is present, the papillae are simple and short [7]. These epithelial characteristics were very similar to those of the present case. In ovarian mucinous tumors, gastrointestinal differentiation that was noted in our case is known to occur more often in borderline tumors and carcinomas rather than benign mucinous tumors. Ovarian intestinal-type mucinous borderline tumors are usually found as a larger size and consist of cysts and glands lined by atypical epithelium of gastrointestinal type [8]. The epithelium is usually stratified to two or three layers, nuclear atypia is mild to moderate, and mitotic figures vary from few to numerous. The papillae are commonly found, and are typically thin, branching, and complex [8]. As a whole, the lining epithelial cells in the present case were distinguished from ovarian borderline mucinous tumors in a degree of epithelial proliferation, cellular atypia, and papillary formation. Mucinous cystadenocarcinomas that typically show definite stromal invasion, more complicated papillary or cribriform epithelial proliferation, and moderate to marked nuclear atypia can be easily ruled out from the present case.

Strictly speaking, it is very rare for us to be aware of any primary tumors of the spermatic cord within the inguinal region that could readily be confused microscopically with papillary cystadenomas. However, when we would take consideration of differential diagnosis, benign papillary mesothelioma or multicystic mesothelioma should be considered firstly under the list of differential diagnosis. This rare tumor of the tunica vaginalis usually appears in young men. Grossly, it consists of a hydrocele sac with papillary or adenomatous excrescences and cystic or solid areas. Microscopically, the tumor is comprised mostly of variably sized papillary formations with fibrovascular cores and covered by cuboidal, columnar, or flattened mesothelial cells with large vesicular nuclei and glassy eosinophilic cytoplasm [9,10], whereas the present case consisted of mucinous epithelial lining and was negative for WT-1, a well-known positive marker of mesothelial cells. Papillary serous tumor of benign or low malignant potential may occur in the tunica vaginalis, testis, spermatic cord, and epididymis. These tumors were described identical to its ovarian counterpart [1,11]. The present case is distinguished by its mucinous epithelial character, bland cytology and no epithelial stratification from serous tumors. Among malignant tumors, mucinous cystadenocarcinoma of this area, if ever, could be easily differentiated from mucinous cystadenoma by typical cytologic and structural anaplasia, including papillary, glandular, mucinous, and solid undifferentiated patterns [12]. Lastly, monodermal and highly specialized teratomas such as endodermal variants of mature cystic teratoma should be also considered [13]. Among them, glandular differentiated (adenomatoid) teratomas are composed mainly of mucinous (intestinal-type) epithelium of endodermal derivation. And these teratomas usually have other minor teratomatous components within the tumors. On the contrary, the present case showed no teratomatous tissue elsewhere in the primary lesion. Instead, it revealed distinct smooth muscle layers encircling around the neoplastic epithelium, which could not be expected from endodermal variants of mature cystic teratoma.

Judging from histologic features, the present case mucinous papillary cystadenoma is a benign, noninvasive lesion that is speculated with no capacity for distant metastasis or recurrence, although its precise natural biology is poorly understood due to their extreme rarity.

## Conclusions

Although clinical follow up observation is relatively short in the present case, the tumor is most likely speculated to be a benign neoplastic process when we combined histological findings with clinical histories (no definite evidence of recurrence during 8-months’follow up, no tumorous lesion identified so far outside inguinal area). Mucinous papillary cystadenoma is an extremely rare neoplastic lesion of the spermatic cord. Clinically, there are no specific symptoms other than palpable mass, and radiologic imaging could give an impression of cystic or metastatic tumor arising in the inguinal region. The desirable treatment of choice is a local complete excision with sparing of the spermatic cord. However, it is important that the nature of these lesions be recognized clinically and pathologically in order to avoid unnecessary radical surgery.

## Consent

Written informed consent was obtained from the patient for publication of this Case Report and any accompanying images. A copy of the written consent is available for review by the Editor-in Chief of this journal.

## Abbreviations

CEA: Carcinoembryonic antigen; CK20: Cytokeratin 20; EMA: Epithelial membrane antigen; WT-1: Wilms’ tumor 1 protein; CDX2: Drosophila caudal-type homeobox 2; PAX8: Paired box 8.

## Competing interests

The authors declare that they have no competing interests.

## Authors’ contributions

JY-KIM carried out the immunohistochemical staining and wrote the most of the manuscript. YT-LEE collected the patient’s clinical information. HJ-KANG, DY-PARK, and GY-HUH participated in pathological investigations and the review of related references. CH-LEE also participated in pathological investigations, revised manuscript for important intellectual content and had given final approval of the version to be published. All of the authors have read and approved the final manuscript.
